# The Evolution of Oligometastatic Pancreatic Ductal Adenocarcinoma: From Anatomic Description to Biologically Informed Patient Selection

**DOI:** 10.3390/cancers18203376

**Published:** 2026-10-19

**Authors:** Kshitij S. Gaur, William A. Hall, Mandana Kamgar, Thomas McFall, Douglas B. Evans, Y. David Seo

**Affiliations:** 1 Department of Surgery, Medical College of Wisconsin, Milwaukee, WI 53226, USA; kgaur@mcw.edu (K.S.G.);; 2 Mary Ann and Charles LaBahn Pancreatic Cancer Program, Medical College of Wisconsin, Milwaukee, WI 53226, USA; 3 Department of Radiation Oncology, Medical College of Wisconsin, Milwaukee, WI 53226, USA; 4 Department of Internal Medicine, Medical College of Wisconsin, Milwaukee, WI 53226, USA; 5 Department of Biochemistry, Medical College of Wisconsin, Milwaukee, WI 53226, USA

**Keywords:** pancreatic ductal adenocarcinoma, oligometastatic disease, metastasis-directed therapy, metastasectomy, stereotactic body radiation therapy, CA19-9, treatment sequencing, patient selection

## Abstract

Metastatic pancreatic ductal adenocarcinoma (PDAC) has historically been managed with systemic therapy, and local therapies have, in general, been avoided in the absence of symptoms directly related to a specific metastasis. Even if a specific site of disease, such as a local recurrence, had the potential to cause symptoms in the future, there was a bias against prophylactic palliation. It was well-established that most patients die of systemic progression. This approach is now under pressure from four directions. Retrospective case series have reported survival durations longer than anticipated in highly selected patients with low-volume, single-organ disease. A randomized phase II trial reported that metastasis-directed therapy prolonged progression-free survival. Biologic data indicate that the site of metastatic relapse is encoded in gene expression profiles already present in the primary tumor. Second-line systemic therapy has become substantially more effective, which lengthens the interval over which control of an untreated metastasis may become clinically important. We trace the oligometastatic concept from the 1995 Hellman and Weichselbaum hypothesis to the 2026 OligoPanc consensus definition of up to three lesions confined to a single organ, examine the criteria proposed for patient selection, summarize the surgical and ablative radiotherapy literature, and consider the trials that will determine whether this is a treatable disease state or a favorable prognostic phenotype. We conclude that induction systemic therapy, followed by response assessment that weighs the absence of disease progression, Carbohydrate Antigen 19-9 (CA19-9) kinetics, and performance status, is a defensible route to local therapy directed at the metastases and the primary tumor, and warrants prospective study.

## Simple Summary

Pancreatic cancer that has spread beyond the pancreas has traditionally been treated with systemic therapy, and patients with distant metastases are rarely considered for local therapies such as surgery or radiation therapy. Over the past decade, however, a subset of patients has repeatedly been identified whose metastases are limited in number or confined to a single organ, whose disease responds to chemotherapy, and whose survival duration has been longer than anticipated. This review traces how the oligometastatic concept has been imported into the field of pancreatic cancer oncology, examines what is now known about how such patients should be identified, and addresses the central question, which is whether treatment directed at metastases changes the natural history of the disease or merely identifies those patients whose biology was favorable from the outset.

## 1. Introduction

The presence of a single distant metastatic deposit is, in most treatment algorithms, sufficient to exclude a patient from any local therapy directed at an anatomic target. For most patients, pancreatic ductal adenocarcinoma (PDAC) disseminates early with subclinical metastatic disease present at the time of diagnosis even when imaging is negative. The dominant mode of death is systemic progression rather than uncontrolled local disease. The application of a local therapy for the treatment of a systemic disease is in contradiction with accepted oncologic principles [1], and the consequences of doing so have been well-documented in the surgical and oncologic literature. However, the same literature has, with increasing consistency over the past fifteen years, produced a residual population that this framework does not accommodate. These are patients with one or two hepatic or pulmonary metastases, a CA19-9 concentration that normalizes on induction chemotherapy, and a survival duration longer than the reported median for metastatic disease.

The existing literature on this population of patients with oligometastatic disease is difficult to interpret because of the use of heterogeneous systemic therapy regimens, inconsistent and largely retrospective definitions of what constitutes oligometastatic disease, selection bias in favor of patients with excellent performance status and likely other favorable variables, and an almost complete absence of randomized comparison. It has therefore remained unclear whether metastasis-directed therapy in PDAC alters the natural history of the disease or whether it functions as an elaborate biomarker identifying patients whose tumors were never going to behave in the way that population-level survival statistics predict. This ambiguity has obvious clinical implications and may determine, for example, whether a patient with a solitary chemotherapy-responsive liver metastasis should be offered a pancreatectomy with synchronous hepatic resection, a course of ablative radiation, or continued systemic therapy alone. These three approaches carry different risks, different treatment-related toxicities, and different degrees of treatment-related immunosuppression.

Our goal in this review is to trace how the oligometastatic concept entered the field of pancreatic cancer therapeutics, to examine the definitions and selection criteria that have been proposed and the evidence supporting each, and to offer a practical framework for treatment sequencing in metastatic PDAC at centers where complex pancreatectomy and ablative radiotherapy are both available. We begin with the origins of the oligometastatic hypothesis and the trials in other disease sites that have established its credibility, then turn to the specific problem of defining the oligometastatic state in PDAC, the biology that may or may not underwrite it, the criteria by which patients should be selected, and the local therapies available. We conclude with an account of what the current evidence does not support and what the trials now accruing hope to show.

## 2. Origins of the Oligometastatic Hypothesis

The proposition that metastatic disease exists along a spectrum rather than as a binary state was advanced by Hellman and Weichselbaum in a 1995 editorial in the Journal of Clinical Oncology [2]. They argued for an intermediate condition, termed oligometastases, in which metastases are limited in number and organ site. Such tumors were believed not to have acquired full metastatic competence and were therefore considered potentially curable by local therapy. This hypothesis was conceptual and claimed that limited metastatic burden reflects a distinct and less aggressive metastatic phenotype rather than an earlier point along the same trajectory. The authors also suggested that treating the visible metastases in such patients could be curative; neither claim was testable with the tools available at that time.

However, two developments brought greater attention to the oligometastatic hypothesis. The first was the maturation of stereotactic ablative radiotherapy, which provided a means of delivering a tumoricidal dose to metastases in multiple organs with acceptable toxicity and without the recovery period that surgical metastasectomy imposed. The second was the accumulation of biologic evidence that the oligometastatic state has molecular correlates. Work from Weichselbaum and colleagues demonstrated that microRNA expression patterns distinguished oligometastatic from polymetastatic disease and that specific microRNA signatures were associated with progression from an oligometastatic to a polymetastatic pattern [3,4]. These data supported the hypothesis that oligometastases are a distinct biological state rather than simply early polymetastases observed at a moment in time.

The initial clinical evidence in support of an oligometastatic state was assembled largely outside of pancreatic cancer. The SABR-COMET trial randomized 99 patients with one to five metastases, from a range of primary sites, to stereotactic ablative radiotherapy or standard palliative care. The investigators reported a median overall survival of 41 months versus 28 months in favor of ablative therapy, a difference that met the trial’s prespecified but unconventional significance threshold. At long-term follow-up, five-year overall survival was 42.3% versus 17.7% [5,6]. In oligometastatic prostate cancer, the STOMP and ORIOLE trials established that metastasis-directed therapy delayed the need for systemic therapy and prolonged progression-free survival [7,8]. A pooled analysis subsequently demonstrated that high-risk mutations in TP53, ATM, BRCA1/2, and RB1 modified the magnitude of that benefit [9]. The SINDAS trial demonstrated a survival advantage for the addition of radiotherapy to first-generation tyrosine kinase inhibitor therapy in synchronous oligometastatic EGFR-mutated non-small cell lung cancer [10]. The CURB trial demonstrated that the benefit of local therapy for oligoprogression was histology-dependent, with a marked progression-free survival advantage in non-small cell lung cancer (10.0 versus 2.2 months; HR 0.41, 95% CI 0.22–0.75; *p* = 0.0039) and no advantage in breast cancer [11].

The colorectal experience with local therapy for metastatic disease is perhaps the most firmly established, although randomized data are less robust. The EORTC 40983 trial randomized 364 patients with resectable colorectal liver metastases to perioperative FOLFOX4 plus surgery or surgery alone. A three-year progression-free survival benefit reached significance only in the trial’s eligible-patient population with four or fewer metastases (HR 0.77, 95% CI 0.60–1.00; *p* = 0.041), and not among all randomized patients (HR 0.79, 95% CI 0.62–1.02; *p* = 0.058) [12]. At a median follow-up of 8.5 years, median overall survival was 61.3 months with perioperative chemotherapy and 54.3 months with surgery alone (HR 0.88, 95% CI 0.68–1.14; *p* = 0.34) [13]. The precedent that pancreatic surgeons most often invoke therefore rests on a progression-free survival signal derived from other primary sites.

The European Society for Radiotherapy and Oncology and the European Organization for Research and Treatment of Cancer subsequently produced a consensus classification that remains the common language of the field [14]. That classification sorts patients along three axes. The first separates genuine oligometastatic disease, which is present before any systemic therapy, from induced oligometastatic disease, in which a larger burden has been reduced by systemic therapy. The second separates a first oligometastatic presentation from a repeat presentation. The third separates synchronous from metachronous timing, and divides the metachronous category further into oligorecurrence (disease that reappears after a disease-free interval), oligoprogression (growth at a limited number of sites while the remainder of the disease is controlled), and oligopersistence (a limited number of sites that never responded). A prospective registry of more than 1000 patients treated under this framework reported one- and three-year local progression rates of 5.0% and 11.4%, with the minimum dose delivered to the planning target volume as the dominant predictor of local control [15]. Two features of the classification matter here. The first is that it is agnostic to the primary site of cancer. The second is that pancreatic cancer was not represented among the studies from which it was derived.

## 3. Why PDAC Was Excluded, and Why That Is Changing

Pancreatic cancer was absent from the early oligometastatic literature for reasons that were understandable. The natural history of metastatic PDAC treated with the systemic therapy available before 2010 was measured in months. The proportion of patients who survived long enough for local control of any lesion to matter was small. The operation required to address a pancreatic primary was not comparable in morbidity to a wedge resection of the lung or liver. Under these circumstances, local therapy directed at any single lesion could not have altered the mode of death.

Since that time, four things have changed. First, multiagent systemic therapy (FOLFIRINOX and gemcitabine with nab-paclitaxel) produced response rates and durations of disease control that had not previously been achievable. In doing so, this created a population of patients in whom systemic disease was controlled for long enough that the fate of individual metastatic deposits became a legitimate clinical question. Second, the widespread adoption of neoadjuvant treatment sequencing for localized, nonmetastatic pancreatic cancer accustomed the field to an adaptive approach, in which the response to induction chemotherapy serves as the most informative staging test available and subsequent treatment decisions are based on the presence or absence of response [1,16,17]. Third, the technical capacity to deliver an ablative dose to abdominal targets improved substantially with the introduction of magnetic resonance-guided online adaptive radiotherapy, which permits daily re-optimization of the treatment plan against the observed position of the target and adjacent luminal organs [18,19,20]. Fourth, and most recently, systemic therapy for metastatic PDAC has become substantially more effective in the second line through the use of pan-RAS(ON) inhibitors. Patients who fail to respond to cytotoxic therapy now have second-line options. Those options lengthen the interval over which local control of individual metastases is clinically meaningful (Section 10).

The question, therefore, is how to define and identify the subgroup of patients in which durable control of a small number of metastases translates into a meaningful extension of survival.

## 4. Defining Oligometastatic PDAC

The field has proposed at least four distinct definitions of oligometastatic PDAC, and the discrepancies among them are the single largest impediment to interpretation of the literature:

Lesion count: The most widely used criterion, and the one adopted by every randomized trial, is the number of metastases. The EXTEND basket trial enrolled patients with five or fewer metastatic lesions [21]. Its PDAC-specific successor EXPAND used one to five, and refined the rule so that each lesion, rather than each site, counted as one, and all metastatic nodal stations collectively counted as a single lesion [22]. The HOLIPANC trial used a maximum of five hepatic metastases [23]. The METAPANC trial used a maximum of three confined to the liver [24], as did the SONAR trial [25]. Lesion count is unambiguous but remains a poor proxy for tumor burden. Five subcentimeter metastases in one patient and three metastases measuring four centimeters in another may have different natural histories.

Organ number and site: The 2026 OligoPanc consensus statement was developed through a modified Delphi process that involved 55 experts from 19 countries, with consensus defined as agreement of at least 75%. The statement defined oligometastatic PDAC as up to three lesions confined to a single organ, either liver or lung [26]. Ninety-eight percent of participants agreed that lesion number was relevant to the definition and 89% agreed that organ number was relevant. The panel accepted both synchronous and metachronous presentations as oligometastatic (76%), with the disease-free interval to be considered in the metachronous setting (78%). The statement failed to reach consensus on absolute CA19-9 or CEA thresholds, lesion size thresholds, the importance of germline testing, or on whether limited peritoneal disease should be included. It also explicitly rejected mandatory peritoneal lavage (84%).

Volumetric burden: Our group has taken a different approach to defining oligometastatic PDAC. In a retrospective analysis of 121 patients with metastatic PDAC treated at our institution with FOLFIRINOX or gemcitabine-based chemotherapy, metastatic disease burden was measured by manually outlining every visible metastasis on the diagnostic computed tomography scan and summing the resulting volumes rather than by counting lesions. That method is referred to here as volumetric segmentation. A landmark analysis was then performed at 18 months [27]. Patients who survived 18 months or longer differed from those who survived less than 18 months in the number of organs involved (solitary versus two or more; *p* = 0.04) and in mean metastatic disease volume (1.4 cc versus 19.1 cc; *p* = 0.04). A total metastatic volume of less than 0.2 cc, or normalization of CA19-9, identified patients whose overall survival was, in the authors’ words, “similar to non-metastatic disease.” Volumetric segmentation measures disease on a continuous scale rather than assigning it to one of a small number of categories, and it separates patients who would be counted identically under any lesion-count definition. It is the most detailed anatomic definition proposed to date, but it requires dedicated radiologic effort that is not yet routine outside of research.

Biochemical and biologic response: The fourth approach abandoned anatomy in favor of response to therapy. Its strongest support came from a series reported from Heidelberg, in which resection after preoperative chemotherapy was termed conversion surgery. After preoperative chemotherapy, 173 patients with metastatic PDAC were explored and 93 underwent resection. Median overall survival was 25.5 months in the 45 patients with a complete pathologic response at metastatic sites (ypM0), compared to 10.7 months in the 48 with residual, viable metastatic disease (ypM1), and 8.1 months in patients not resected (*p* < 0.001) [28]. The ypM0 versus ypM1 difference was a factor of more than two, within a cohort that was anatomically identical at the time of the decision to operate. It is the strongest evidence in the literature that response to systemic therapy, rather than anatomic burden, correlates most closely with clinical benefit. Unfortunately, ypM0 status can only be determined after the operation has been performed, which is a distinct disadvantage for those with persistent metastatic disease.

The definitions of oligometastatic PDAC described above are applied to three populations of patients that are often discussed together. The first is initial oligometastatic disease, termed genuine oligometastatic disease in the European Society for Radiotherapy and Oncology-European Organisation for Research and Treatment of Cancer (ESTRO–EORTC) classification, in which a small number of metastases is present at diagnosis, before any systemic therapy. The second is induced oligometastatic disease, in which a patient who had more extensive metastatic disease at diagnosis has a small number of remaining sites after systemic therapy [14]. The third is the population selected for surgery on the basis of response to systemic therapy, in which the decision to operate rests on a favorable biochemical and radiographic response rather than on lesion count. Patients from either of the first two populations may enter the third. The Heidelberg data described above suggest that this distinction is important [28]. Because every patient in the framework proposed here receives systemic therapy first (Section 6), the population that reaches local therapy is selected by response to systemic therapy, regardless of the initial anatomic burden.

Table 1 summarizes the evolution of the four definitions. Our position is that the OligoPanc consensus should be used for trial eligibility and cross-study comparison, because a common language is worth more at this stage than a perfect one. Volumetric burden and CA19-9 kinetics should be captured alongside lesion count in every prospective cohort, so that the relative predictive weight of each can eventually be determined.

## 5. Is There a Biology of Oligometastatic Pancreatic Cancer?

The oligometastatic hypothesis suggests that limited metastatic burden reflects a distinct biological state. In PDAC, the evidence for such a state is suggestive but incomplete.

Prior work has established that metastatic competence in PDAC is acquired late. Yachida and colleagues used multi-region sequencing of matched primaries and metastases from rapid autopsy specimens to demonstrate that metastatic clones genetically evolved from a parental non-metastatic clone. They suggested that a decade elapsed between the initiating mutation and the emergence of the parental founder cell, and that at least five additional years were required before metastases emerged [31]. These observations describe, at least in principle, a window during which dissemination is limited in extent. That window is the mechanistic premise on which the oligometastatic hypothesis depends.

It is also established that the site of metastatic relapse is not random. Paget proposed in 1889, and Fidler subsequently restated and extended, the seed and soil hypothesis, which holds that a metastasis develops only where the disseminated tumor cell and the receiving organ are compatible [32]. The site of metastasis also appears to influence the behavior of the disease. In a study of 744 patients who underwent resection of PDAC, the 100 patients whose first recurrence was isolated to the liver had significantly worse overall survival than the 31 whose first recurrence was isolated to the lung. Single-nucleus RNA sequencing of the primary tumors showed that tumors from patients who later recurred in the liver or the lung expressed gene expression profiles resembling those of normal liver and normal lung parenchymal cells, respectively. No genetic alteration predicted the site of recurrence [33]. Organotropism appears to be encoded in pre-existing cellular states within the primary tumor. Consistent with this, the lung-first phenotype has repeatedly been shown to carry a survival advantage, and lung-only and liver-only metastatic PDAC differ in molecular profile [34,35,36]. George and colleagues identified four microenvironment classes with distinct therapeutic implications in a transcriptomic classification of the pancreatic tumor microenvironment, providing a framework in which such organ-specific differences can be interpreted [37].

In contrast, no published work has established that these organ-specific gene expression profiles constitute a signature of oligometastatic disease in PDAC. The microRNA studies described above, which support a distinct oligometastatic state, were performed in other primary tumor types [3,4]. The transcriptional subtypes of PDAC (classical and basal-like in the Moffitt schema; squamous, pancreatic progenitor, immunogenic and ADEX in the Bailey schema; and classical, quasimesenchymal and exocrine-like in the Collisson schema) may have prognostic and therapeutic relevance, but they have not been shown to identify an oligometastatic phenotype [38,39,40]. Recent spatial transcriptomic studies indicated that subtype assignment is plastic rather than fixed, which argues against a subtype-based selection strategy [41]. The authors of the OligoPanc consensus statement stated that there is “no evidence in PDAC to support a more refined definition incorporating genomic, transcriptomic, or epigenomic signatures” [26]. In summary, the primary tumor in PDAC carries gene expression profiles that correspond to the organ in which metastases later appear, and those profiles correlate with survival. Whether they also determine the number of metastases, which is the principal claim of the oligometastatic hypothesis, remains untested. Liquid biopsy offers a means of testing whether limited metastatic burden reflects limited dissemination. Detectable circulating tumor DNA at baseline was associated with shorter progression-free survival in the EXTEND aggregate analysis (Section 9) [42], and the OLIGOMETS trial requires undetectable circulating tumor DNA for eligibility [30].

## 6. Guiding Principles for Patient Selection

The following principles are offered as a framework for prospective evaluation rather than as recommendations supported by randomized evidence. In general, surgery should be limited to carefully selected patients using objective criteria, and not because other therapies have been exhausted [43]. Each principle is labeled by the strength of its support. An evidence-supported principle rests on published data, although not on randomized trials in patients with metastatic PDAC. Institutional practice describes our approach where data are lacking. A hypothesis requires prospective validation.

Principle 1 (evidence-supported principle): All patients with distant metastases receive systemic therapy first; patients are not selected for local therapy at the time of diagnosis. Eighty-two percent of the OligoPanc panel endorsed systemic therapy before local consolidative therapy for patients with synchronous metastases [26]. The same rationale has governed our approach to localized, nonmetastatic PDAC: induction chemotherapy identifies patients in whom the visible disease is representative of the disease as a whole, rather than the leading edge of a much larger occult burden [1,16,17,44]. Selection should be applied before the operation, not discovered afterward. A patient whose disease progresses on induction therapy has been identified as a patient who would not have benefited, and this outcome represents the framework working rather than failing.

Principle 2 (evidence-supported principle): Biochemical response is more sensitive than radiographic response. The unreliability of radiographic response assessment in PDAC is among the best-documented observations in the field. Two mechanisms account for it. The dense stroma of PDAC forms a scaffold that may prevent a measurable decrease in tumor size even when there has been substantial cell death. In addition, the relationship between the tumor and the adjacent vessels, which is the principal determinant of resectability, rarely changes even when tumor size does decrease [43]. In 129 patients with borderline resectable disease treated with neoadjuvant therapy, 122 were restaged; 15 (12%) had a partial response by RECIST and 84 (69%) had stable disease. Only 1 (0.8%) of the 129 patients was reclassified as resectable, yet 85 (66%) underwent pancreatectomy. Among the patients who underwent pancreatectomy, survival bore no relationship to RECIST category (*p* = 0.78) [45,46]. CA19-9, by contrast, performs well. Normalization of CA19-9 after neoadjuvant therapy in localized, nonmetastatic disease is associated with a median overall survival of 46 versus 23 months (*p* = 0.02) [47]. Its positive predictive value for reaching an operation is high, but its negative predictive value is 28–33%; a persistently elevated CA19-9 therefore identifies a patient unlikely to benefit but does not by itself justify withholding local therapy [48]. Pretreatment CA19-9 has no such predictive value, so it is the change upon therapy and not the baseline that carries information [49]. Additionally, CA19-9 response to first-line and second-line regimens can be used to guide regimen switching [50], and in the metastatic setting, CA19-9 normalization was the dominant predictor of two-year survival in our own cohort (*p* = 0.001) [27]. Absolute value, normalization, percentage reduction, and serial kinetics are distinct measures, and none has been prospectively validated as a selection criterion in patients with metastatic PDAC. We regard a sustained decline on serial measurements as the minimum requirement for biochemical response. Normalization is the most favorable finding, but it is not sufficient on its own to select a patient for local therapy. A reduction of at least 50% from baseline is our institutional convention. Approximately 30% of patients are Lewis antigen-negative and do not produce CA19-9, and the marker is not interpretable when the total bilirubin exceeds 2 mg/dL, which is a common circumstance in this population. Patients with obstructive jaundice should therefore undergo biliary decompression, and the baseline CA19-9 should be recorded after the bilirubin has normalized [51]. Machine learning-derived surrogate tumor markers have also been developed at our institution to extend biochemical response assessment to patients who do not produce CA19-9 [52,53]. Fluorodeoxyglucose positron emission tomography also provides complementary information on metabolic response and is endorsed by the OligoPanc consensus panel for use in unclear cases (82%) [26,54].

Principle 3 (hypothesis requiring prospective validation): Anatomic burden should be measured, not counted. Lesion count is the currency of trial eligibility, but organ number and total metastatic volume correlated more closely with survival in our own cohort [27]. Small indeterminate hepatic lesions are common at diagnosis and are frequently benign. Characterizing them correctly is a prerequisite to any claim about oligometastatic status [55]. Staging chest computed tomography identifies pulmonary disease that alters the treatment plan [56]. Computed tomography is also insensitive for regional nodal metastases. In a prospective comparison with pathology, a 10 mm size criterion identified 1 of 7 malignant nodes, and 2 of 23 nodes smaller than 5 mm contained metastatic disease [57]. Regional nodes that appear normal on imaging, including nodes along the superior mesenteric artery, may therefore contain metastatic disease at pathologic examination. We do not perform laparoscopy at the time of diagnosis in the absence of a specific indication. It is performed at the time of anesthesia induction for a planned resection, or earlier when imaging or the CA19-9 concentration raises a specific concern for peritoneal disease, in which setting it identifies low-volume peritoneal disease that would make any consolidative strategy futile [58]. In a prospective series of locally advanced, nonmetastatic disease from our own institution, metastatic disease was identified at operation in 7 (15%) of 47 patients [59]. Performance status and medical comorbidity should be re-assessed at restaging and weighed as seriously as anatomy, since a patient who has not tolerated induction therapy is unlikely to tolerate a pancreatectomy combined with hepatic or pulmonary resection [1,60]. Nutritional status should be assessed at the same time, and a dietitian should be part of the multidisciplinary team, because nutritional assessment and support are essential for patients being prepared for pancreatic resection [61].

Principle 4 (institutional practice): The interval of systemic therapy before local consolidation should be long enough to be informative. There is no trial-derived answer to the question of how long induction therapy should continue before local therapy is considered, and we are unaware of any published data that identify an optimal duration in patients with metastatic PDAC. The METAPANC trial used eight cycles of mFOLFIRINOX before randomization [24], which introduced an obvious selection bias. There is a second and separate reason to wait. An interval of systemic therapy spares the patient whose disease is already progressing the morbidity of a local therapy that cannot help, and this matters most when the local therapy itself carries appreciable morbidity, as irreversible electroporation does. The duration should therefore be sufficient for the pattern of failure to declare itself. Restaging at intervals of six to eight weeks, after four cycles of mFOLFIRINOX or two cycles of gemcitabine with nab-paclitaxel, is the practice we have applied to localized, nonmetastatic disease [16,62]. Non-response is treated as an indication to change regimens rather than to abandon local therapy, and dose intensity and regimen quality matter more than cycle number [62]. The competing toxicities of prolonged therapy, biliary events chief among them in this population, impose a practical ceiling that is frequently reached before the theoretical optimum [63].

Principle 5 (institutional practice): Local consolidative therapy should be delivered only at centers where both surgical and radiotherapeutic options are available, and only after multidisciplinary review. Ninety-three percent of the OligoPanc panel considered multidisciplinary board review essential [26], and the National Comprehensive Cancer Network guideline conditions it explicitly on referral to a high-volume center [64]. The choice between synchronous metastasectomy and ablative radiotherapy is a clinical judgment, and a program that can offer only one of them may make that judgment poorly. We would emphasize, however, that a case-by-case multidisciplinary decision is not by itself a criterion. The criteria we would apply (lesion number, volumetric burden, CA19-9 response, performance status, and technical feasibility at every site of disease) should be stated in advance and recorded prospectively, because generalizability to less experienced centers rests on codification rather than on expertise [43]. A recommendation to refer is not by itself a solution. Referral to high-volume centers has been advocated for decades without a corresponding change in where these operations are performed [65], and the obstacles are structural rather than clinical: narrow insurance networks, closed delivery systems, distance, and institutional pressure to retain patients. What can be implemented now is to separate the decision from the operation. Review of the diagnostic imaging, the CA19-9 kinetics, and the performance status by a program that performs both pancreatectomy and ablative radiotherapy can be obtained remotely, with systemic therapy continued at the referring center. Recording that review, and reporting the proportion of patients for whom it occurred, converts an exhortation into a process that can be measured.

## 7. Local Consolidative Therapy: Surgery

The surgical literature in oligometastatic PDAC is entirely retrospective, and the most important number is the cohort denominator. Frigerio and colleagues reported that 24 (4.5%) of 535 patients with metastatic PDAC were ultimately taken to resection after a response to induction therapy. Among those 24 patients, an R0 resection was achieved in 88% and a complete pathologic response in 17%. Median overall survival was 56 months and median disease-free survival was 27 months [66]. Those survival durations are longer than any other series has reported, but they were observed in 24 patients. Any series that has reported outcomes of metastasectomy in PDAC has selected patients through three sequential filters: referral to a high-volume center, survival through months of systemic therapy, and the subjective assessment that the operation is worth performing. In patients with localized, nonmetastatic pancreatic cancer, our group published stage-specific probabilities of completing all intended neoadjuvant therapy and surgery: 90%, 75%, 60% and 25% for resectable, borderline resectable, locally advanced type A and locally advanced type B disease, respectively. Such data allow physicians and patients to establish goals of therapy at the time of diagnosis [60]. No series of resection for oligometastatic PDAC has reported the equivalent probability. The Verona denominator is the closest the literature comes, and every prospective cohort in this population should report it. Survival measured from the start of systemic therapy in patients who later underwent resection includes the months of induction therapy that these patients had to survive in order to become candidates for surgery. That interval is a source of immortal time bias. A causal benefit of surgery cannot be inferred without a nonsurgical comparator selected at the same landmark time, and every series should report the outcome of the entire initial cohort of patients with metastatic disease, not only of those who underwent resection (Section 13).

The published series are consistent with one another, subject to the selection described above (Table 2). A report from Heidelberg included 128 patients who underwent radical resection of oligometastatic PDAC. Median overall survival after M1 resection was 12.3 months. Morbidity was 45% and 30-day mortality was 2.9%; 90-day mortality was not reported. Five-year survival was 8.1% after resection of liver metastases and 10.1% after resection of interaortocaval nodal disease [67]. A multicenter analysis matched 69 patients who underwent synchronous hepatic resection to 69 who underwent exploration without resection, and reported a median overall survival of 14 versus 8 months in favor of resection (*p* < 0.001) [68]. Hank and colleagues, in the series discussed above, demonstrated that the survival benefit was concentrated in patients with a complete pathologic response of metastases [28]. A nationwide multicenter study from the Japan Pancreas Society included 90 patients with liver metastases at diagnosis, including occult metastases found at operation, who underwent conversion surgery after at least four months of chemotherapy. The median duration of preoperative chemotherapy was 10.4 months, and liver metastasectomy was performed in 27 (30%) of the 90 patients. Median overall survival was 53.1 months from the start of treatment and 39.7 months after surgery. Preoperative normalization of CA19-9 and pathologically negative lymph nodes were independent predictors of overall survival [69]. These findings support biochemical response, rather than anatomic burden at diagnosis, as the basis for selection. Survival measured from the start of treatment in that study also illustrates the denominator problem described above, because only patients who completed at least four months of chemotherapy and were then selected for surgery were included. A systematic review of the surgical literature concluded that resection “may be feasible, safe and associated with prolonged survival in selected patients.” The reviewers identified response to chemotherapy, low tumor marker levels, limited metastatic number, and complete macroscopic resection as the favorable factors, and noted that patients with isolated pulmonary metastases fared particularly well [70]. These reports are, however, retrospective and subject to considerable selection bias. The strongest form of that objection has been stated by our own group in the context of locally advanced, nonmetastatic disease. Patients selected on the basis of response to induction therapy will likely realize a significant survival benefit even without an operation [43].

Isolated pulmonary disease deserves separate treatment. Pulmonary recurrence after resection of PDAC is associated with markedly longer survival than hepatic recurrence, both in single-institution series and in secondary analyses of adjuvant trials [34,71,72], and this observation now has a mechanistic correlate in the organotropism data discussed above [33]. Whether pulmonary metastasectomy contributes to that survival, or whether the lung-first phenotype simply identifies favorable biology that would have been favorable regardless, is not resolved by any published dataset. We interpret the association cautiously, as it almost certainly reflects the selection for local therapy of patients with limited disease burden, favorable biology, and preserved performance status.

The international position papers that address surgery in this setting must be read with attention to their scope. The 2022 International Association of Pancreatology–Japan Pancreas Society consensus on conversion surgery reached unanimous agreement that “cancer biology is surpassing locoregional anatomical resectability” [73]. That document addresses locally advanced, nonmetastatic disease. Metastatic disease is addressed instead by its companion position paper on resection for PDAC with distant abdominal organ metastasis [74].

## 8. Local Consolidative Therapy: Ablative Radiotherapy

The argument for radiotherapy rather than surgery in this setting is straightforward. Metastasis-directed radiation can be delivered to hepatic and pulmonary targets and to the pancreatic primary without a laparotomy, does not interrupt systemic therapy for the weeks that recovery from a pancreatectomy requires, and can be repeated. In the EXTEND aggregate analysis, radiotherapy was used for 370 (98%) of the 379 lesions treated with metastasis-directed therapy [42]. That proportion reflects how metastasis-directed therapy is currently delivered. Where both modalities are available, whether the patient is a candidate for resection should be settled before radiotherapy is planned, rather than in parallel with it [75].

Our institution’s position on local control in PDAC has been stated repeatedly and does not depend on the oligometastatic question. Local disease control matters most in patients whose systemic disease is controlled, because a patient must be alive to benefit from it. The dominant threat remains the development of further metastatic disease. Local therapy should therefore be applied selectively to patients who are responding to systemic therapy [75]. The field’s drift away from radiation in pancreatic cancer has been driven in part by trials whose radiotherapy was not designed to achieve local control [76,77].

The technical basis for ablative treatment of abdominal targets is now established. Magnetic resonance-guided online adaptive radiotherapy permits daily plan re-optimization against the observed anatomy, and our group’s early experience with liver metastases, primary hepatic malignancy, and pancreatic cancer on a 1.5 Tesla MR-linac established the feasibility of this approach [18,19,20,78]. In borderline resectable and locally advanced disease, the SMART trial delivered 50 Gy in five fractions to 136 patients. Two-year overall survival from diagnosis was 53.6%, with acceptable late toxicity [79]. That trial did not enroll patients with metastatic disease, and its results should not be presented as though it had.

Percutaneous thermal ablation offers a nonradiotherapeutic alternative for small hepatic metastases. In 60 patients with metachronous hepatic metastases after resection of PDAC (median diameter 1.5 cm), radiofrequency ablation was technically successful in all patients. Major complications occurred in 8 (13.3%) of the 60 patients, and the rate of local tumor progression was 41.2% at one year [80]. The HOPE-1 trial combines thermal hepatic ablation with resection of the primary tumor [81].

## 9. The Randomized Evidence in Oligometastatic PDAC

The randomized evidence specific to oligometastatic PDAC comes from the pancreas basket of EXTEND, in which 41 patients with five or fewer metastases were randomized to metastasis-directed therapy plus systemic therapy or systemic therapy alone. Among the 40 analyzed (19 versus 21), median progression-free survival was 10.3 months (95% CI 4.6–14.0) with metastasis-directed therapy versus 2.5 months (95% CI 1.7–5.1) with systemic therapy alone (HR 0.43, 95% CI 0.20–0.94; *p* = 0.030). No grade 3 or higher adverse events were attributed to the local therapy, and there was evidence of systemic immune activation that correlated with improved progression-free survival [21]. EXTEND is the only randomized trial of metastasis-directed therapy that has enrolled patients with PDAC. Its limitations are clear. It was a 40-patient randomized phase II study with a progression-free survival endpoint and was not powered for overall survival. In addition, the control-arm median progression-free survival of 2.5 months is considerably shorter than contemporary first-line systemic therapy would be expected to deliver. The subsequently reported aggregate analysis of all six EXTEND baskets, comprising 334 per-protocol patients at a median follow-up of 53 months, reported a progression-free survival hazard ratio of 0.54 (95% CI 0.41–0.72; *p* < 0.001) overall and 0.60 (95% CI 0.40–0.89) excluding the prostate baskets. Detectable circulating tumor DNA at baseline correlated with shorter progression-free survival, and clearance by three months correlated with improved survival [42].

The pancreas-specific successor to EXTEND is now accruing. EXPAND (Extending Outcomes for Pancreas Cancer Patients With Nominal Oligometastatic Disease, NCT06593431) is a randomized trial at the same institution, restricted to PDAC rather than structured as a basket. Patients with one to five distant metastatic lesions amenable to local therapy at every site are randomized 1:1 to metastasis-directed therapy plus systemic therapy, or to systemic therapy alone [22]. Its lesion-counting rule is the most precise definition yet written into an oligometastatic PDAC protocol. Each lesion counts as one, rather than each site. Metastatic lymph node stations collectively count as a single lesion, and bilateral nodal chains count as separate sites. Malignant effusion, leptomeningeal disease, and peritoneal carcinomatosis are excluded.

## 10. Systemic Therapy for Metastatic PDAC Has Changed

Any framework that conditions local therapy on the response to systemic therapy depends on how effective that systemic therapy is, and that premise changed in 2026. Daraxonrasib is an orally bioavailable RAS(ON) multi-selective inhibitor that binds cyclophilin A and engages the active, guanosine triphosphate-bound state of both mutant and wild-type RAS, sterically blocking effector binding [82]. In the phase 1/2 experience, 168 patients with previously treated RAS-mutated PDAC received doses up to 300 mg daily. Among the 26 patients with RAS G12-mutant disease treated in the second line at the 300 mg dose, the objective response rate was 35% (95% CI 17–56), median progression-free survival was 8.5 months, and median overall survival was 13.1 months [83]. RASolute 302 was a randomized phase III trial of daraxonrasib versus investigator’s choice of chemotherapy in 500 patients with previously treated metastatic PDAC. Median overall survival was 13.2 versus 6.7 months (HR 0.40, 95% CI 0.30–0.53) and median progression-free survival was 7.2 versus 3.6 months (HR 0.49, 95% CI 0.38–0.64). The objective response rate was 30% versus 11%, and there were fewer grade 3 or higher adverse events in the experimental arm [84]. The agent was approved by the United States Food and Drug Administration in August 2026, and the European Society for Medical Oncology issued an express guideline update recommending daraxonrasib monotherapy after progression on chemotherapy in patients with RAS G12-mutated PDAC and performance status 0 or 1 [85].

**Figure 1:**
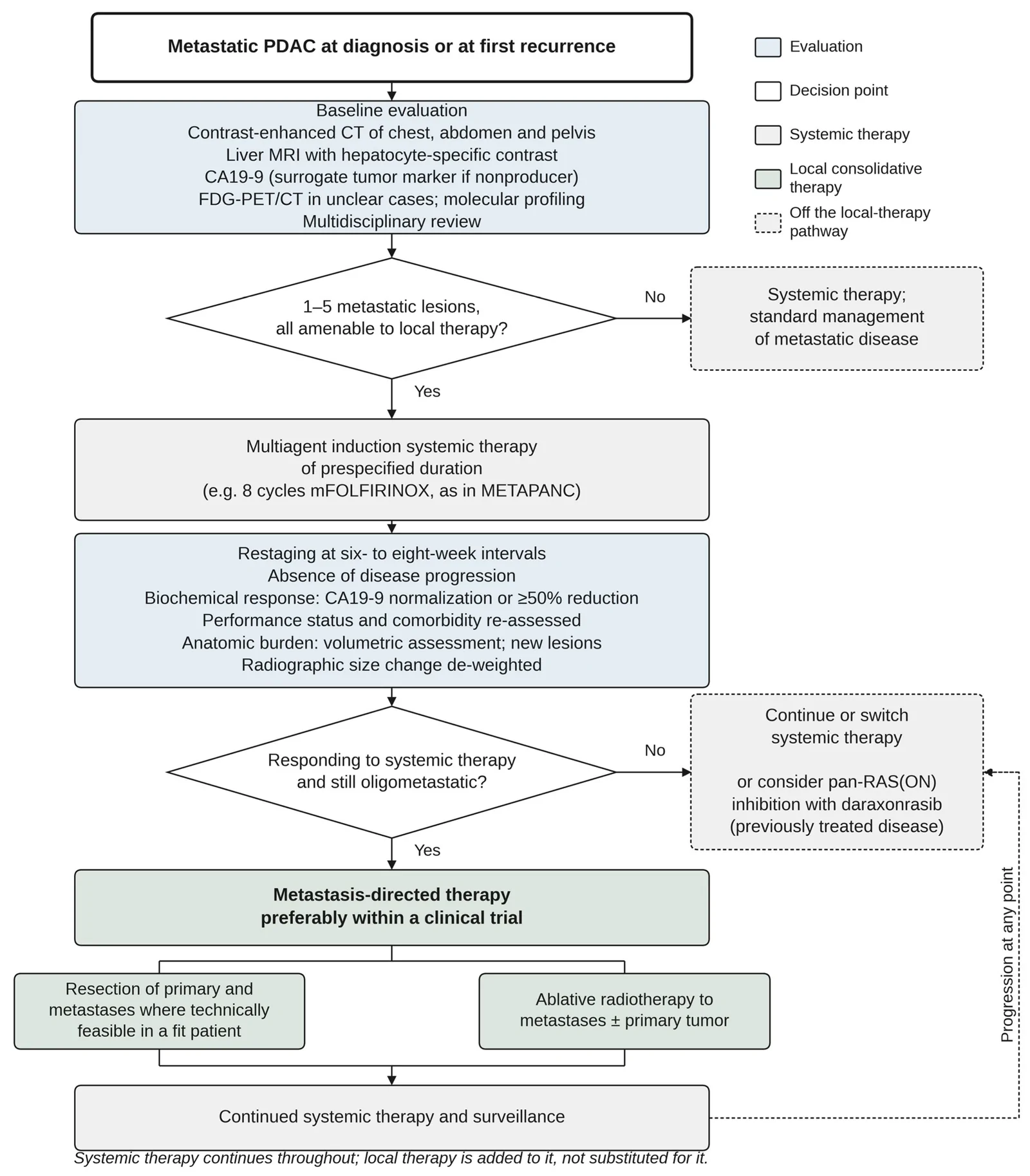
Proposed treatment-sequencing algorithm for metastatic pancreatic ductal adenocarcinoma with limited metastatic burden. All patients undergo baseline contrast-enhanced computed tomography of the chest, abdomen, and pelvis, hepatocyte-specific contrast magnetic resonance imaging of the liver, CA19-9 measurement (with a surrogate tumor marker in nonproducers), and multidisciplinary review. Patients meeting anatomic criteria (one to five metastatic lesions, all amenable to local therapy, the threshold used by EXTEND and EXPAND [21,22]) proceed to multiagent induction systemic therapy of defined duration. Restaging at six- to eight-week intervals assesses absence of disease progression, biochemical response (CA19-9 normalization or ≥50% reduction), performance status and medical comorbidity, and anatomic burden (volumetric assessment, appearance of new lesions). Patients with biochemical and anatomic response are evaluated for local consolidative therapy (synchronous resection where technically feasible in a fit patient, ablative radiotherapy otherwise), preferably on a clinical trial. Patients who do not respond, whether to initial therapy or at any subsequent point (dashed return path), continue or switch systemic therapy, or are considered for pan-RAS(ON) inhibition with daraxonrasib, which prolonged survival against investigator’s choice chemotherapy in previously treated metastatic disease [84]. Patients who respond and remain oligometastatic proceed to metastasis-directed therapy and then to continued systemic therapy with surveillance.

This matters to the oligometastatic question in three ways, none of which has been formally tested. First, a second-line median overall survival of 13.2 months roughly doubles the interval during which local failure of an untreated metastasis could become clinically consequential, which strengthens rather than weakens the rationale for durable local control. Second, it changes what should happen to the patient who does not meet response criteria on induction therapy. Continuation of an ineffective regimen is no longer the only alternative to local therapy, and a genotype-directed change in regimen is now a reasonable next step in RAS G12-mutant disease (Figure 1). Third, the trial program has moved into the micrometastatic and first-line settings, with RASolute 304 in resected PDAC after neoadjuvant or adjuvant chemotherapy and RASolute 303 in the first line. No registered trial combines daraxonrasib with metastasis-directed therapy, and none enrolls a population defined as oligometastatic. This is an addressable gap for future study.

## 11. Trials in Progress

Table 3 summarizes the prospective trials that address oligometastatic PDAC, together with the outcomes reported to date. Questions over the use of surgery rest on four randomized trials, METAPANC, SONAR, CSPAC-1, and HOPE-1, and three single-arm prospective studies, HOLIPANC, ScanPan 1, and PHOLIPANC. METAPANC is an international phase III trial in which patients received eight cycles of mFOLFIRINOX. Responding patients with three or fewer liver-confined metastases were then randomized to resection of the primary tumor and metastases with perioperative chemotherapy, or to continued chemotherapy alone. The primary endpoint was overall survival, and the target was 272 patients; 60 patients had been randomized as of March 2026 [24]. SONAR randomized patients with up to three synchronous liver metastases to resection versus observation or continued chemotherapy, after at least six months of first-line chemotherapy with response or stable disease and a falling or minimally rising CA19-9. The primary endpoint was the two-year overall survival rate [25]. CSPAC-1, conducted by the Chinese Study Group for Pancreatic Cancer, randomized patients with hepatic oligometastasis after conversion chemotherapy to simultaneous resection of the primary and metastases versus standard therapy [86]. HOPE-1, at Zhejiang University, was designed to randomize patients with three or fewer liver lesions of 3 cm or less, or a single lesion of 5 cm or less, to resection of the primary tumor with thermal hepatic ablation and chemotherapy, or to chemotherapy alone. The primary endpoint was overall survival and the target was 60 patients. Its registry record lists it as not yet recruiting and was last updated in 2022 [81]. HOLIPANC was a single-arm phase II trial. Patients with five or fewer liver metastases received neoadjuvant liposomal irinotecan with oxaliplatin and 5-fluorouracil/folinic acid, with resection in responders and comparison against historical controls [23]. Interpretation of that design will be limited by the changes in staging, imaging and systemic regimens that separate any contemporary cohort from its historical comparator. At the time of writing, HOLIPANC’s estimated primary and overall completion dates have passed without posted results, and the CSPAC-1 registry record has not been updated since 2018 against a primary completion date of 2023. ScanPan 1, a Scandinavian prospective cohort of multimodal treatment for patients with up to four liver metastases responding to four months of induction chemotherapy, has a published 2025 protocol but a registry record last updated in 2022 [29]. PHOLIPANC, which is still described as ongoing in parts of the secondary literature, was terminated after enrolling five patients [87]. None of these trials has reported results, and we emphasize that the METAPANC material presented to date is a trials-in-progress report and not a readout.

The radiotherapy question is addressed by EXPAND, discussed above [22], and by four further trials in which patients with PDAC are eligible. OligoRARE, an EORTC phase III trial, randomized patients with one to five metastases from primary sites other than prostate, breast, lung, and colorectum to standard care with palliative radiotherapy versus standard care with stereotactic body radiotherapy, with overall survival as the primary endpoint [88]. The SENECA trial randomized patients with first-line metastatic pancreatic cancer to chemotherapy with or without radiotherapy directed at the primary and, where the disease is oligometastatic, at distant lesions. The primary endpoint of SENECA is pain control rather than survival, and its registry record specifies no numeric lesion threshold [89]. A Korean single-arm phase II trial evaluated personalized ultrafractionated stereotactic adaptive proton therapy in borderline resectable, locally advanced, or oligometastatic disease defined as three or fewer lesions [90]. A Chinese single-arm phase II trial evaluated nimotuzumab with chemoradiotherapy in unresectable locally advanced or oligometastatic disease using the same threshold [91].

Two further trials are worth noting. The PANFIRE-3 trial combined irreversible electroporation with nivolumab and an intratumoral Toll-like receptor–ligand, and defined oligometastatic disease without a lesion threshold, leaving the maximum to be set case-by-case by the multidisciplinary tumor board. This is an explicit acknowledgement that the numeric definitions are arbitrary. Its registry status is unknown and its completion date has passed [92]. The OLIGOMETS trial, which opened in 2026, randomized patients with metastatic foregut adenocarcinoma, PDAC included, to sequential cytoreductive intervention versus standard care, and gated eligibility on undetectable circulating tumor DNA rather than on anatomy alone [30].

**Figure 2:**
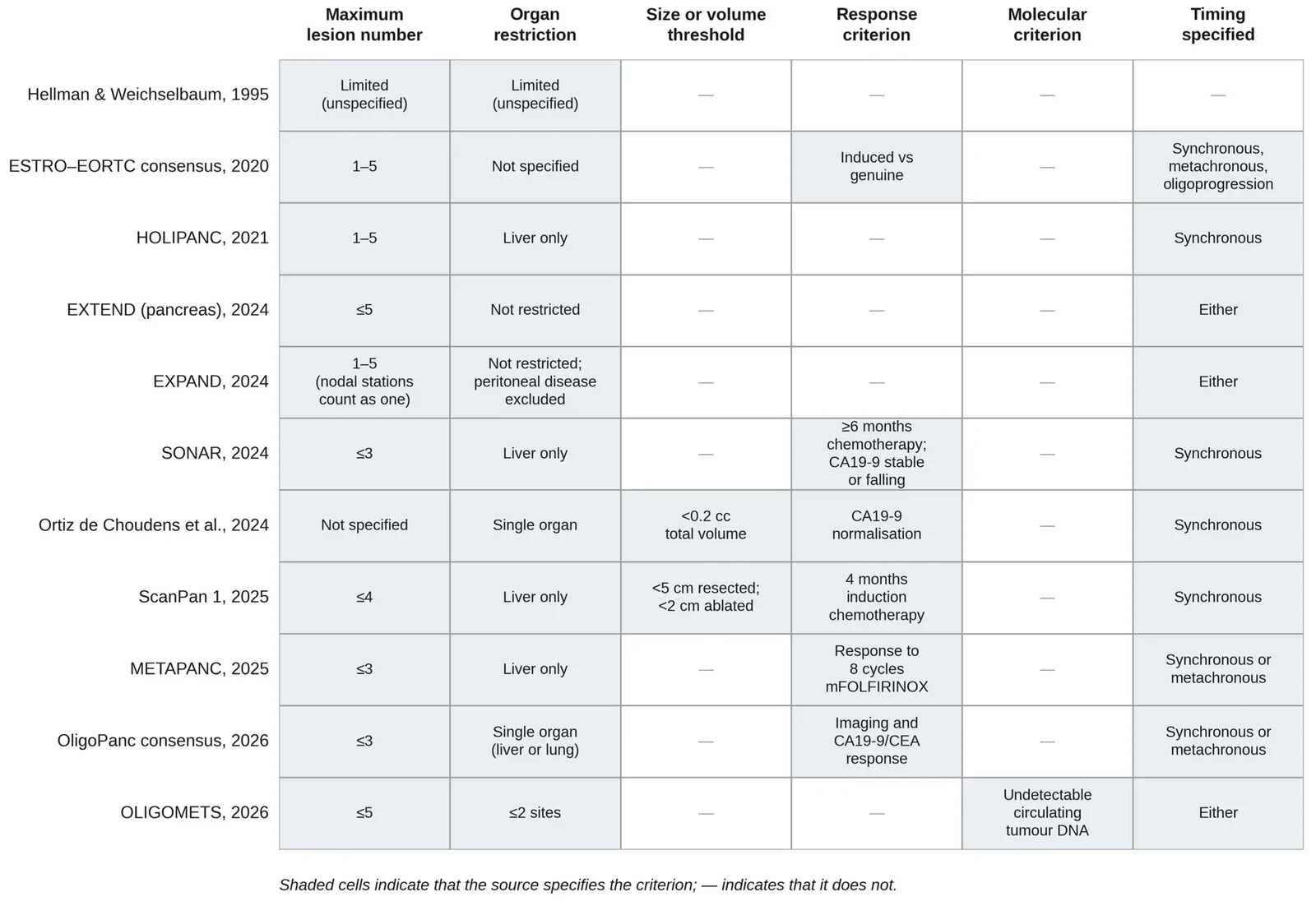
Divergence of published definitions of the oligometastatic state in pancreatic ductal adenocarcinoma, with shaded cells indicating that the source specifies and defines the classification scheme [2,14,21,22,23,24,25,26,27,29,30]. This illustrates the overlapping, but non-identical populations captured by lesion-count, organ-confinement, volumetric, and response-based criteria. The 2026 consensus threshold of three lesions in one organ is narrower than the eligibility criteria of most trials currently accruing, including EXPAND, SONAR, ScanPan 1 and OLIGOMETS.

Finally, we would like to draw attention to a structural mismatch. The OligoPanc consensus defined oligometastatic PDAC as three or fewer lesions confined to one organ [26], but the trials that will report over the next five years are not enrolling to that definition. For example, EXTEND and EXPAND permit up to five lesions in any organ, HOLIPANC one to five, ScanPan 1 up to four, and OLIGOMETS up to five across two sites, while only METAPANC, SONAR, CSPAC-1, and the proton and nimotuzumab trials use a threshold of three. The consensus definition and the trials that will test it are therefore drifting apart before either is settled (Figure 2). The discrepancy should be resolved deliberately, rather than by whichever trial reports first.

## 12. What the Current Guidelines Say

The National Comprehensive Cancer Network guideline for pancreatic adenocarcinoma (Version 2.2025) does not recommend resection of the primary tumor or metastasectomy as standard management of metastatic PDAC, but does state that “in rare circumstances when patients have indolent/oligometastatic disease, they should be referred to a high-volume center to be evaluated for possible metastatic directed therapy.” Metastasis-directed therapy is listed as an optional addition in the maintenance setting [64]. The 2026 American Society for Radiation Oncology clinical practice guideline conditionally recommends radiotherapy to metastatic lesions and to the primary tumor for patients with oligometastatic and oligoprogressive pancreatic cancer [93]. The American Society of Clinical Oncology guideline for metastatic pancreatic cancer addresses systemic therapy and molecular testing but does not address local therapy for oligometastatic disease [94]. The current European Society for Medical Oncology guideline [95] and its 2026 express update on daraxonrasib address systemic therapy only [85]. The OligoPanc consensus statement remains the only document dedicated to this population [26].

## 13. Where Evidence Remains Lacking

Several gaps in the current literature define the work that remains. Each is stated below together with the study design that would close it.

First, every published retrospective surgical series in oligometastatic PDAC is subject to selection bias at multiple sequential stages, and selection alone could be responsible for the reported results. The Verona denominator described in Section 7 reported the survival of the 24 patients who underwent resection, which may not reflect the effect of the operation [66]. The ypM0 versus ypM1 comparison within the Heidelberg series [28] is the closest study available to an internal control, and even there the assignment is post hoc. The remedy is available and inexpensive. Every series should report the number of patients with metastatic PDAC evaluated at the outset, the number who began induction therapy, and the number who underwent local therapy, so that the survival reported can be placed against the population from which it was drawn.

Second, immortal time bias is intrinsic to this body of literature. Patients must have survived induction chemotherapy, restaging, and referral in order to become candidates for local therapy. Comparisons to patients treated with systemic therapy alone from the time of diagnosis are structurally biased in favor of local therapy unless landmark or time-varying methods are used; few published series do so. Both methods are well-established and should be expected of any future comparison.

Third, the definitions are not interchangeable between published reports. A trial enrolling five or fewer lesions in any organ [21], a consensus definition of three or fewer lesions in one organ [26], a volumetric threshold of 0.2 cc [27], and an eligibility rule gated on undetectable circulating tumor DNA [30] all describe overlapping but distinct populations of patients. Recording lesion number, organ number, volumetric burden, and circulating tumor DNA status prospectively would allow each cohort to be reported against all four definitions and would make series comparable without waiting for the field to agree on one of them.

Fourth, the prospective evidence is more fragile than published trial lists suggest. Of the trials registered to answer the question of whether surgery prolongs survival, one was terminated after enrolling five patients [87], two have registry records that have not been updated against completion dates that have passed [23,86], one has a published protocol but a dormant registration [29], and one has not opened [81]. Reviews that list these trials as ongoing may overstate how much randomized evidence is actually accruing. Registry records should be updated at the intervals the registries require, and reviews should state the date on which each record was last updated.

Fifth, the randomized evidence consists of the 40-patient phase II EXTEND trial with a progression-free survival endpoint [21]. Its hazard ratio is impressive, but its confidence interval is wide, and the biological plausibility of the finding does not substitute for adequately powered confirmation. An adequately powered randomized trial with an overall survival endpoint is the single most valuable contribution now available and EXPAND is designed to provide it [22].

Sixth, the outcomes reported by high-volume, multidisciplinary programs, including our own, should not be extrapolated to centers without comparable surgical and radiotherapeutic expertise. The higher survival and resection rates reported at such centers reflect a concentration of expertise, prospective application of response-driven treatment sequencing, mitigation techniques for many of the medical challenges involved in caring for patients with pancreatic cancer, and referral patterns that select favorably at diagnosis. Metastasis-directed therapy for PDAC is therefore best delivered at high-volume centers, preferably within a clinical trial. As discussed in Section 6, a recommendation to refer has not by itself changed where these operations are performed. What can be implemented now is a remote review of the diagnostic imaging, the CA19-9 kinetics, and the performance status by a program that offers both resection and ablative radiotherapy, with systemic therapy continued at the referring center and the review recorded in the medical record [43,65]. The proportion of patients for whom such a review occurs can be measured and should be reported.

Finally, we would note that the field lacks any validated molecular signature of the oligometastatic state in PDAC, that the optimal duration of systemic therapy before local consolidation is unknown, that no CA19-9 threshold has been prospectively validated for this indication, and that whether the pancreatic primary should be resected, irradiated, or left alone in a patient whose metastases have been ablated is entirely unstudied. Each of these is a defined and answerable question, and the framework proposed here should be regarded as hypothesis-generating rather than as a standard of care.

## 14. Future Directions and Conclusions

Three lines of work may materially advance the field. The first is the completion of the METAPANC and SONAR trials, which will establish whether the surgical signal survives randomization, and of the EXPAND trial, which advances the EXTEND trial experience into a PDAC-specific randomized design [22]. None of these trials addresses whether metastasis-directed therapy should be combined with RAS(ON) inhibition [84]. The second is the prospective collection of biomarkers that are not yet gathered routinely. These include volumetric metastatic burden rather than lesion count; CA19-9 and other tumor-associated antigens, including E19-9 as recently reported by Thalji and colleagues [52,53]; circulating tumor DNA and associated molecular tumor fragments [42,96]; and transcriptomic classification of the tumor microenvironment of the primary tumor [37]. In combination, these could outperform anatomy as a selection variable. The third is the systematic characterization of the biology of organ-specific metastatic relapse [33], and specifically whether the gene expression profiles that determine the site of metastasis also constrain the number of metastases. That is the question on which the oligometastatic hypothesis in PDAC ultimately rests. Prospective integration of tumor molecular profiling with volumetric and biochemical response criteria therefore warrants evaluation in larger, multi-institutional cohorts with linked tissue banking, and molecularly informed trial designs in metastatic PDAC are already demonstrating what such integration can yield [97,98,99].

In summary, oligometastatic pancreatic ductal adenocarcinoma has evolved over three decades from a conceptual proposition with no evidentiary basis into a defined clinical entity with a consensus definition and a randomized signal of benefit from metastasis-directed therapy. Treatment of oligometastatic PDAC has an emerging biological rationale, grounded in organ-specific gene expression profiles present in the primary tumor, and two randomized trials of surgery, METAPANC and SONAR, together with the randomized EXPAND trial of metastasis-directed therapy, are accruing. However, the central question the concept poses remains unanswered: whether treating the visible metastases with local ablative therapy alters the natural history of the disease or merely identifies the patients whose disease was never going to follow the expected course. At present, local ablative therapy for metastatic PDAC should be applied only after an adequate interval of systemic therapy to patients with stable or responding disease (absence of radiographic progression, CA19-9 response, and adequate performance status), and, wherever possible, as part of a clinical trial.

## Figures and Tables

**Table 1:** Evolution of definitions of oligometastatic disease relevant to pancreatic ductal adenocarcinoma.

Source	Year	Population	Definition	Basis
Hellman & Weichselbaum [2]	1995	All solid tumors	Conceptual: metastases limited in number and organ site, reflecting incomplete metastatic competence	Hypothesis
ESTRO–EORTC consensus [14]	2020	All solid tumors	Classification framework: genuine vs. induced; de novo vs. repeat; synchronous vs. metachronous (oligorecurrence, oligoprogression, oligopersistence)	Delphi consensus
EXTEND trial [21]	2024	Basket, incl. PDAC	≤5 metastatic lesions	Trial eligibility
Ortiz de Choudens et al. [27]	2024	Metastatic PDAC	Solitary organ involvement; total metastatic volume < 0.2 cc; CA19-9 normalization	Retrospective, volumetric segmentation
METAPANC [24]	2025	Metastatic PDAC	≤3 liver-confined metastases	Trial eligibility
HOLIPANC [23]	2021	Metastatic PDAC	≤5 liver-confined metastases	Trial eligibility
OligoPanc consensus [26]	2026	PDAC	≤3 lesions confined to a single organ (liver or lung); synchronous or metachronous	Modified Delphi, 55 experts, ≥75% threshold
EXPAND trial [22]	2024	PDAC only	1–5 distant metastatic lesions, all amenable to local therapy; each lesion (not site) counts as one, all nodal stations collectively as one; peritoneal carcinomatosis excluded	Trial eligibility
SONAR [25]	2024	Metastatic PDAC	≤3 synchronous liver metastases; no extrahepatic disease	Trial eligibility
ScanPan 1 [29]	2025	Metastatic PDAC	≤4 liver metastases (<5 cm if resected, <2 cm if ablated); no extrahepatic disease	Trial eligibility
OLIGOMETS [30]	2026	Foregut adenocarcinoma, incl. PDAC	≤2 metastatic sites and ≤5 lesions, plus undetectable circulating tumor DNA	Trial eligibility

**Table 2:** Selected series of resection for metastatic pancreatic ductal adenocarcinoma.

Series	*n*	Setting	Median OS	Key Qualifier
Hackert et al. [67]	128	Radical resection of oligometastatic PDAC	12.3 mo after M1 resection	Morbidity 45%; 30-day mortality 2.9%; 5 yr survival 8.1% (liver), 10.1% (interaortocaval nodes)
Tachezy et al. [68]	69 vs. 69 (matched)	Synchronous hepatic resection vs. exploration	14 vs. 8 mo (*p* < 0.001)	Retrospective multicenter
Frigerio et al. [66]	24 of 535 (4.5%)	Resection after downstaging	56 mo (DFS 27 mo)	R0 88%; pCR 17%; the 4.5% denominator is the essential context
Hank et al. [28]	93 resected of 173 explored	Conversion surgery after chemotherapy	25.5 mo (ypM0) vs. 10.7 mo (ypM1) vs. 8.1 mo (no resection), *p* < 0.001	Benefit concentrated in complete pathologic response of metastases

**Table 3:** Prospective trials in oligometastatic pancreatic ductal adenocarcinoma, with reported progression-free and overall survival where available. Status as of 1 September 2026. MDT, metastasis-directed therapy; ST, systemic therapy; NR, not reported; NA, not applicable.

Trial (Identifier)	Design, Size, and Oligometastatic Definition	Intervention	Status	Median PFS	Median OS
EXTEND, pancreas cohort (NCT03599765) [21]	Randomized phase II basket; 40 analyzed (19 vs. 21); ≤5 lesions, organ unrestricted; primary endpoint PFS	MDT + ST vs. ST	Reported 2024; trial active, not recruiting	10.3 mo (95% CI 4.6–14.0) vs. 2.5 mo (95% CI 1.7–5.1); HR 0.43 (95% CI 0.20–0.94), *p* = 0.030	Not powered for OS; NR
EXTEND, all baskets (NCT03599765) [42]	Aggregated analysis; 334 per protocol (166 vs. 168); median follow-up 53 mo; ≤5 lesions	MDT + standard of care vs. standard of care	Reported 2026	HR 0.54 (95% CI 0.41–0.72), *p* < 0.001; HR 0.60 (95% CI 0.40–0.89) excluding prostate baskets	NR
EXPAND (NCT06593431) [22]	Randomized phase III, PDAC only; target *n* = 80; 1–5 distant lesions all amenable to local therapy, nodal stations counted collectively as one; primary endpoint PFS	MDT + ST vs. ST	Recruiting; opened Oct 2024, primary completion Jun 2029	NR	NR
METAPANC (EU CT 2023-503558-10-00) [24]	Randomized phase III; target *n* = 272; ≤3 resectable or ablatable liver metastases; primary endpoint OS	8 cycles mFOLFIRINOX → resection of primary and metastases + 4 cycles vs. 12 cycles chemotherapy	Recruiting; 60 randomized as of Mar 2026	NR	NR
SONAR (NCT06690528) [25]	Randomized phase II; *n* = 56; ≤3 synchronous liver metastases, no extrahepatic disease; primary endpoint 2-year OS rate	Resection of primary and liver metastases vs. observation or continued chemotherapy	Recruiting; opened Nov 2024	NR	NR
CSPAC-1 (NCT03398291) [86]	Randomized phase III; target *n* = 300; ≤3 liver metastases; primary endpoint OS	Simultaneous resection after conversion chemotherapy vs. standard chemotherapy	Uncertain; registry not updated since 2018, primary completion date (2023) passed	NR	NR
HOLIPANC (NCT04617457) [23]	Single-arm phase II; target *n* = 150; 1–5 liver metastases, synchronous only; primary endpoint OS after R0/R1 resection	Neoadjuvant NAPOX → resection	Uncertain; completion dates passed without posted results	NR	NR
ScanPan 1 (NCT05271110) [29]	Prospective single-arm cohort; *n* = 64; ≤4 liver metastases, no extrahepatic disease; primary endpoints safety and feasibility	4 months induction chemotherapy → resection or ablation	Uncertain; protocol published 2025, registry last updated 2022	NR	NR
PHOLIPANC (NCT06122480) [87]	Single-arm phase II; synchronous hepatic or pulmonary metastases, not both	FOLFIRINOX → synchronous resection	Terminated; actual enrollment 5	NA	NA
HOPE-1 (NCT05611359) [81]	Randomized; *n* = 60; ≤3 liver lesions ≤ 3 cm, or a single lesion ≤ 5 cm; primary endpoint OS	Resection of primary + thermal hepatic ablation + chemotherapy vs. chemotherapy	Not yet recruiting; registry last updated 2022	NR	NR
OligoRARE (NCT04498767) [88]	Randomized phase III, EORTC; target *n* = 200; 1–5 metastases; PDAC eligible as a non-prostate, breast, lung or colorectal primary; primary endpoint OS	Standard care + SBRT vs. standard care + palliative radiotherapy	Recruiting	NR	NR
SENECA (NCT06946420) [89]	Randomized phase III; *n* = 108; no numeric lesion threshold in the registry record; primary endpoint pain control	First-line chemotherapy ± radiotherapy to primary and oligometastatic lesions	Active, not recruiting	NR	NR
PULSAR proton therapy (NCT07269626) [90]	Single-arm phase II; *n* = 47; ≤3 lesions; primary endpoints 1-year PFS and 2-year local control	Personalized ultrafractionated stereotactic adaptive proton therapy after 2–4 cycles of systemic therapy	Recruiting	NR	NR
Nimotuzumab + chemoradiotherapy (NCT07386704) [91]	Single-arm phase II; *n* = 30; ≤3 lesions; primary endpoint 1-year local control	Nimotuzumab + radiotherapy or SBRT + S-1 or capecitabine	Enrolling by invitation	NR	NR
PANFIRE-3 (NCT04612530) [92]	Phase I, three arms; *n* = 18; no numeric threshold, maximum set case-by-case by the tumor board; primary endpoint safety	Irreversible electroporation + nivolumab ± intratumoral TLR ligand	Unknown; completion date passed	NR	NR
OLIGOMETS (NCT07282912) [30]	Randomized phase II; *n* = 54; ≤2 metastatic sites and ≤5 lesions and undetectable circulating tumor DNA; primary endpoint PFS	Sequential cytoreductive intervention vs. standard of care	Recruiting; opened Jun 2026	NR	NR

## Data Availability

No new data were created or analyzed in this study. Data sharing is not applicable to this article.
